# Modeling and characteristic analysis of roadway profile under the influence of multiple factors

**DOI:** 10.1038/s41598-022-24205-6

**Published:** 2022-11-18

**Authors:** Zhixiang Liu, Kang Zou, Miao Xie, Chunxue Xie, Zhan Sun

**Affiliations:** 1 Research Institute of Mineral Resources Development and Utilization Technology and Equipment, Liaoning Technical University, Fuxin, 123000 Liaoning Province China; 2 School of Mechanics and Engineering, Liaoning Technical University, Fuxin, 123000 Liaoning Province China; 3 Present Address: School Of Mechanical Engineering, Liaoning Technical University, Fuxin, Liaoning Province China

**Keywords:** Engineering, Mechanical engineering

## Abstract

Considering that the movement trajectory of roadheader cutting head directly affects the surface morphology characteristics of roadway forming, the creation mechanism of roadway contour obtained by cutting head cutting roadway is analyzed. The robot kinematics analysis method was used to determine the coordinates of the roadheader cutting head in the roadway space coordinate system, and the mathematical model of the cutting head cutting the roadway contour was constructed. Through numerical calculation, the differences of roadway morphology characteristics formed by three different types of cutting heads are analyzed. The orthogonal experiment was designed, and the regression equation under multiple factors was solved with the experimental results. The influence of cutting feed rate, cutting Angle, cutting head radius and cutting head cone Angle on the morphology characteristics of cutting surface was analyzed by regression calculation and theoretical calculation. The research results show that the roadway surface obtained by "spherical crown + cylinder" cutting head is the most uneven, and the roadway surface obtained by "spherical crown + cone + cylinder" cutting head is the most flat. With the decrease of cutting lifting Angle, cutting feed, cutting head cone Angle and the increase of cutting head crown radius, the smoothness of roadway obtained by cutting is higher.

## Introduction

Roadway surface topography is directly affected by the cutting head trajectory of roadheader. It is found that the propagation of wireless communication electromagnetic wave in tunnel is directly affected by tunnel surface roughness. For example, when cutting the coal wall, the longitudinal swing and lateral swing of the cutting arm will form two different roadway profiles. In addition, the geometric profile of the cutting head and the cutting feed have a direct impact on the roadway surface profile. The larger the surface roughness of roadway is, the greater the loss of communication electromagnetic wave is, and the higher the frequency of electromagnetic wave is, the more serious the influence will be^1^. The axial velocity of the main section is directly proportional to the surface roughness. The thickness of the low wind speed zone increases, and eddy current zone is easy to form in the backflow zone. The more uneven the roadway surface roughness is, the larger the eddy current zone in the roadway will be^2^. In addition, the contact stiffness between advanced support equipment and surrounding rock of comprehensive excavation is also affected by roadway surface roughness, and thus the dynamic characteristics of advanced support equipment during operation will also be affected^3^. Advanced excavation roadway tends to lead to uneven thickness of shotcrete structure layer in initial support, thus affecting mechanical properties of support structure and stability of surrounding rock^4^.

It can be seen from the above that the roadway surface roughness should be reduced as much as possible when the roadheader cuts the roadway. At present, many scholars have done a lot of research on the dynamics, kinematics and forming control of roadheader^5–16^, which will not be repeated in this paper. For the cutting head of roadheader, scholars have conducted a series of studies on the tooth shape and tooth distance of the cutting head to explore the cutting performance and cutting efficiency^17–19^. In terms of the control and motion analysis of the cutting head, literature^20^ proposes an adaptive control method to improve the cutting speed of the cutting head of the roadheader by using multi-sensor information, aiming at the problems of low cutting efficiency and lack of intelligent control of the roadheader in the process of tunneling. Through the geometric analysis and pose description of the outer envelope surface of the simplified cutting head, literature^21^ elaborated the factors affecting the shape and size of the roadway section. Although a large number of scholars have conducted in-depth research on roadheader and its cutting head in many aspects, there is little research on the surface topography characteristics of roadheader cutting roadway. In this context, the surface topography characteristics of roadheader with some sections are studied in this paper, which provides a basis for cutting parameter optimization and high-precision forming control method of roadheader, and provides a theoretical basis for solving the urgent problem of reducing the surface roughness of roadheader.

This paper is devoted to the modeling of cutting head movement trajectory, mathematical description of roadway surface contour cut by cutting head, and simulation of roadway exterior contour. The forming mechanism of the outer contour of roadway in the process of cutting roadway by cutting head is discussed.Then, the trajectory of the cutting head is analyzed from the Angle of geometric motion, including the geometric description of the contour of the cutting head and the transformation of the moving coordinate system.

This article through the use of robot kinematics analysis method, the precise solution of the roadheader cutting head trajectory, at the same time, the surface contour of roadway is described mathematically. The influence of different cutting head structure size and driving process parameters on the topography information of the outer contour of roadway is analyzed, and then the selection of reasonable parameters has a certain guiding significance for reducing the roughness of the outer contour of roadway surface.

## Roadway surface forming process under the influence of cutting head

Roadway surface forming is a complex process, which is mainly affected by geometric factors generated by roadheader kinematics and cutting phenomenon generated by coupling dynamics between cutting head and coal rock. Geometrical factors refer to the factors that affect the forming morphology of roadway surface from the Angle of relative movement between cutting head and coal rock. For example, the position of the roadheader, the lifting Angle of the cutting part, the geometric size of the cutting head, the cutting feed and so on. Cutting phenomenon refers to vibration and plastic deformation of coal and rock during cutting. Due to cutting phenomenon involving the whole machine system and coupling characteristics of surrounding rock, is very complex and many random factors, it is difficult to accurately quantitative analysis, this article first from the effects of the geometry factors to build the roadway outer contour cutting head cutting mathematical model and simulation model, analysis of different process parameters on the cutting head structure size and mine excavation roadway outside the influence law of the morphology of contour information.

**Figure 1:**
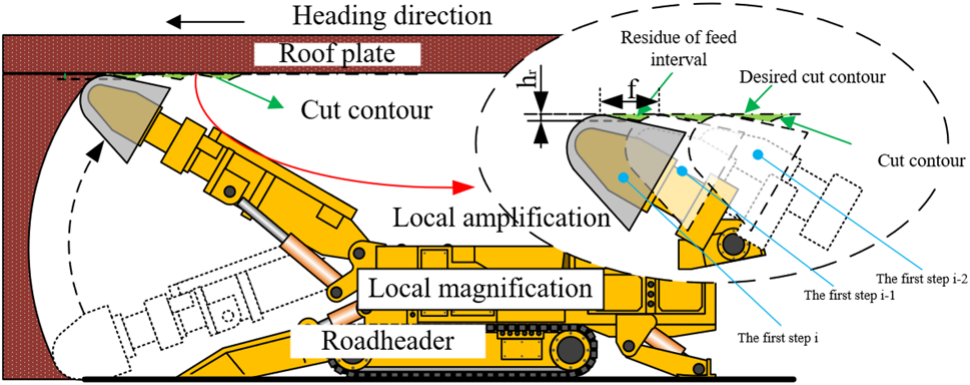
Schematic diagram of roof forming process of driving roadway.

The forming mechanism of roadway surface topography should be studied before the mathematical model and simulation model of workpiece outer roadway contour are developed from the influence of geometric factors. The forming process of roadway roof is shown in Fig. 1.

In Fig. 1, h_f_ is the residual height of the feed interval. By analyzing the cutting feed residues (green filling in the figure) at the first and first steps, it can be seen that each cutting will form a "pit" on the coal rock, and there is a uplift "peak ridge" between each adjacent "pit". The geometric characteristics of "pits" and "ridges" are related to the cutting Angle, the geometric size of the cutting head and the shape of the cutting head. Coal and rock under two adjacent cut feed residues constitute feed spacing residues. The surface texture is formed by the regular distribution of residual coal and rock in 3d space, and its undulation forms the 2d microscopic contour.

In summary, it can be concluded that the cutting feed, the lifting Angle of the cutting part, the shape of the cutting head, the radius of the cutting head and the cone Angle of the cutting head determine the height characteristics and regional distribution characteristics of the residual coal rock after the cutting roof, which are important parameters affecting the surface morphology of the cutting.

## Kinematics model of cutting head

Most studies show that the geometric movement of the cutting part greatly affects the regional distribution and height characteristics of the residual coal rock. Therefore, analyzing the movement of cutting head relative to roadway is the key to reveal the formation mechanism of roadway surface topography. The following is the kinematics model construction of the cutting part and the trajectory analysis of the cutting head.

### Definition of kinematics coordinate system of cutting head

**Figure 2:**
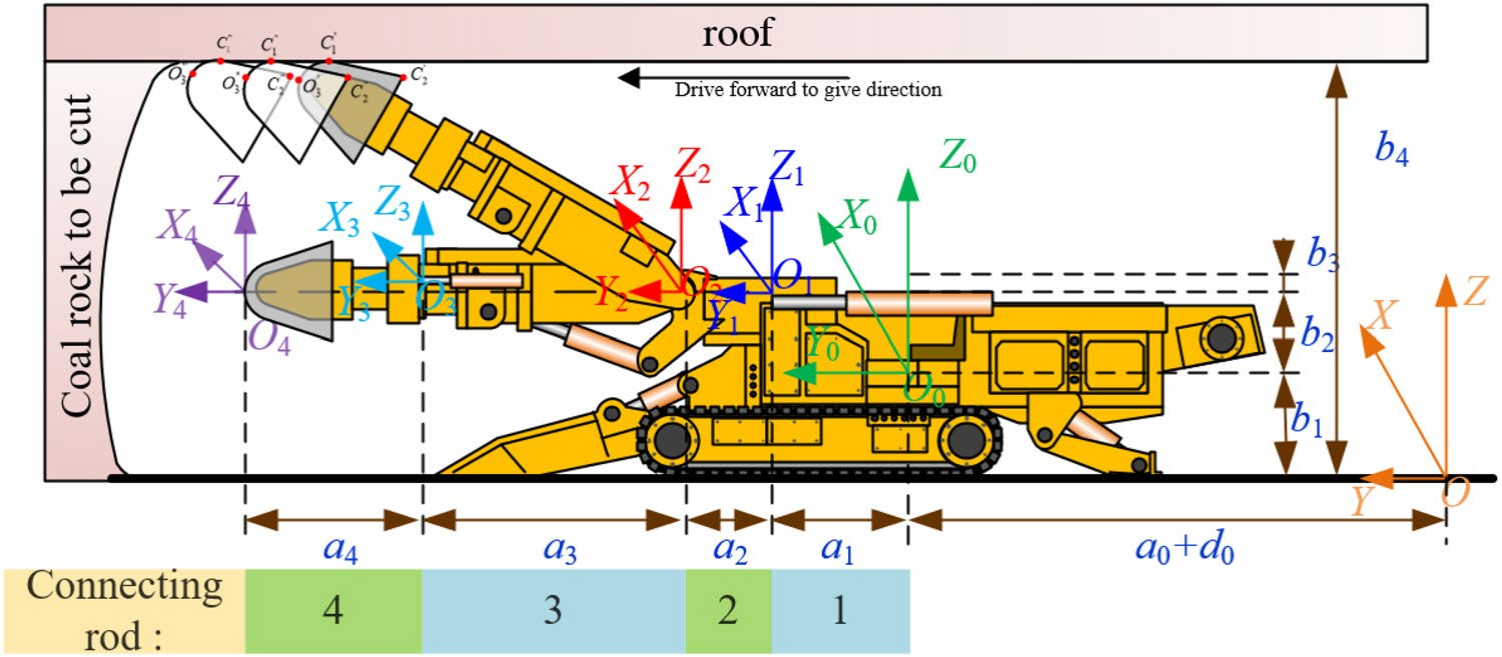
Kinematic coordinate system of roadheader.

The kinematics coordinate system of roadheader is shown in Fig. 2. O_0_X_0_Y_0_Z_0_ is the coordinate system of roadheader, OXYZ is the space coordinate system of roadway, OiXiYiZi is the coordinate system of each mechanism of roadheader. i1–i4 are rotary table coordinate system, cutting part coordinate system, cutting part telescopic part coordinate system, cutting head coordinate system respectively. After the above coordinate system is determined, the motion and pose relationship of the cutting head of roadheader in the roadway space can be obtained.

The kinematic system of the machine is an open-loop system, and the movement of the cutting head in the cutting roadway is controlled by the joint action of the rotary platform, the lifting of the cutting part, the movement of the telescopic part and the movement of the body of the roadheader in the roadway. In the figure, θ1 is the horizontal rotation Angle of the rotary platform, θ2 is the vertical lifting Angle, D is the displacement of the expansion part, and D0 is the cutting feed. In this paper, D-H hair in robotics is used to carry out kinematic analysis on the cutting head of roadheader^15,16^. The position of each point on the cutting head can be transformed into roadway space by describing the pose of each mechanism of the roadheader. In the coordinate system of roadway space, the secondary matrix of coordinate transformation of cutting head is:

1 $$\begin{aligned} {}_{4}{\text{T}} & = {}_{0}{\text{T}}_{1}^{0} {\text{T}}_{2}^{1} {\text{T}}_{3}^{2} {\text{T}}_{4}^{3} {\text{T}} \\ & = \left[ {\begin{array}{*{20}l} {\cos \theta _{1} } \hfill & { - \sin \theta _{1} \cos \theta _{2} } \hfill & {\sin \theta _{1} \sin \theta _{2} } \hfill & { - \left( {a_{3} + a_{4} + d} \right)\sin \theta _{1} \cos \theta _{2} - a_{2} \sin \theta _{1} } \hfill \\ {\sin \theta _{1} } \hfill & {\cos \theta _{1} \cos \theta _{2} } \hfill & { - \cos \theta _{1} \sin \theta _{2} } \hfill & {\left( {a_{3} + a_{4} + d} \right)\cos \theta _{1} \cos \theta _{2} + a_{2} \cos \theta _{1} + a_{1} + a_{0} + d_{0} } \hfill \\ 0 \hfill & {\sin \theta _{2} } \hfill & {\cos \theta _{2} } \hfill & {\left( {a_{3} + a_{4} + d} \right)\sin \theta _{2} + b_{2} + b_{1} + b_{3} } \hfill \\ 0 \hfill & 0 \hfill & 0 \hfill & 1 \hfill \\ \end{array} } \right] \\ \end{aligned}$$

$$\begin{aligned} & {\text{Type}}\;_{0} {\mathbf{T}} = \left[ {\begin{array}{*{20}l} 1 & 0 & 0 & 0 \\ 0 & 1 & 0 & {a_{0} + d_{0} } \\ 0 & 0 & 1 & {b_{1} } \\ 0 & 0 & 0 & 1 \\ \end{array} } \right];\;_{1}^{0} {\mathbf{T}} = \left[ {\begin{array}{*{20}l} {\cos \theta _{1} } & { - \sin \theta _{1} } & 0 & 0 \\ {\sin \theta _{1} } & {\cos \theta _{1} } & 0 & {a_{1} } \\ 0 & 0 & 1 & {b_{2} } \\ 0 & 0 & 0 & 1 \\ \end{array} } \right]; \\ & \quad {}_{2}^{1} {\mathbf{T}} = \left[ {\begin{array}{*{20}c} 1 & 0 & 0 & 0 \\ 0 & {\cos \theta _{2} } & { - \sin \theta _{2} } & {a_{2} } \\ 0 & {\sin \theta _{2} } & {\cos \theta _{2} } & {b_{3} } \\ 0 & 0 & 0 & 1 \\ \end{array} } \right];\;_{3}^{2} {\mathbf{T}} = \left[ {\begin{array}{*{20}l} 1 & 0 & 0 & 0 \\ 0 & 1 & 0 & {a_{3} + d} \\ 0 & 0 & 1 & 0 \\ 0 & 0 & 0 & 1 \\ \end{array} } \right];\;_{4}^{3} {\mathbf{T}} = \left[ {\begin{array}{*{20}l} 1 & 0 & 0 & 0 \\ 0 & 1 & 0 & {a_{4} } \\ 0 & 0 & 1 & 0 \\ 0 & 0 & 0 & 1 \\ \end{array} } \right]. \\ \end{aligned}$$

The coordinate vector of the center of the cutting head in the cutting head coordinate system is ^4^p = [0 0 0 1]^T^, which can be converted to the coordinate system of the roadway by the following formula:

2 $${\mathbf{p}} = {}_{4}^{{}} {\mathbf{T}} \cdot {}^{4}{\mathbf{p}} = \left[ {x\,{\kern 1pt} {\kern 1pt} y{\kern 1pt} {\kern 1pt} z{\kern 1pt} {\kern 1pt} {\kern 1pt} 1} \right]^{{\text{T}}}$$

Among them, *x* = − (*a*_3_ + *a*_4_ + d) sin *θ*_1_ cos *θ*_2_ − *a*_2_ sin *θ*_1_; *y* = (*a*_3_ + *a*_4_ + d) cos *θ*_1_ cos *θ*_2_ + *a*_2_ cos *θ*_1_ + *a*_1_ + *a*_0_ + *d*_0_; *z* = (*a*_3_ + *a*_4_ + d) sin *θ*_2_ + *b*_2_ + *b*_1_ + *b*_3_.

### Cut surface contour model

Accurate mathematical description of roadway, establishment of accurate roadway mathematical model and simulation of cutting head movement model are the basis of roadway surface topography characteristics research. In this process, the most critical is to find the common ground between the cutting head movement model and the tunnel mathematical model. The intersection of the two models is considered as the cut surface contour model.

**Figure 3:**
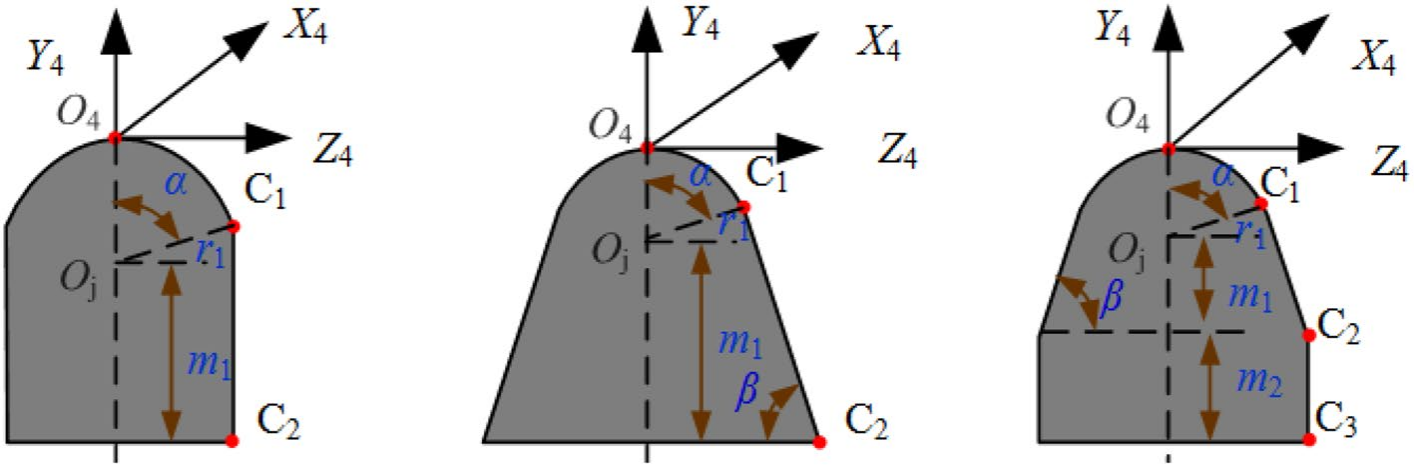
Type and coordinate definition of cutting head.

At present, the common forms of cutting head of roadheader are "ball crown + cylinder" type, "ball crown + cone" type and "ball crown + cone + cylinder" type three types, among which "ball crown + cone + cylinder" type cutting head is most commonly used. Key nodes O4, Oj, C1, C2 and C3 were selected on the cutting head, and the coordinate definition of key nodes on the cutting head type and section was shown in Fig. 3.

According to the geometric size of "spherical crown + cylindrical" cutting head, it can be known that the coordinate vectors of O_4_, Oj, C_1_ and C_2_ in the cutting head coordinate system are ^4^**p**_**O4**_ = [0 0 0 1]^T^, ^4^**p**_**Oj**_ = [0 − *r*_1_ 0 1]^T^, ^4^**p**_**C1**_ = [0 − *r*_1_(1 − cos *α*) *r*_1_ sin *α* 1]^T^, ^4^**p**_**C2**_ = [0 − *r*_1_ − *m*_1_ *r*_1_ sin *α* 1]^T^.

According to the geometric size of "spherical cap + conical" cutting head, it can be known that the coordinate vectors of O4, Oj, C1 and C2 in the cutting head coordinate system are ^4^**p**_**O4**_ = [0 0 0 1]^T^, ^4^**p**_**Oj**_ = [0 − *r*_1_ 0 1]^T^, ^4^**p**_**C1**_ = [0 − *r*_1_(1 − cos *α*) *r*_1_ sin *α* 1]^T^, ^4^**p**_**C2**_ = [0 − *r*_1 _− *m*_1_ *r*_1_ sin *α* + (*m*_1_ + *r*_1_ cos *α*)/tan *β*]^T^.

According to the geometric size of the "spherical cap + cone + cylinder" cutting head, it can be known that the coordinate vectors of O4, Oj, C1, C2 and C3 in the cutting head coordinate system are ^4^**p**_**O4**_ = [0 0 0 1]^T^, ^4^**p**_**Oj**_ = [0 − *r*_1_ 0 1]^T^, ^4^**p**_**C1**_ = [0 − *r*_1_(1 − cos *α*) *r*_1_ sin *α* 1]^T^, ^4^**p**_**C2**_ = [0 − *r*_1 _− *m*_1_ *r*_1_ sin *α* + (*m*_1_ + *r*_1_ cos *α*)/tan *β*]^T^, ^4^**p**_**C3**_ = [0 − *r*_1 _− *m*_1 _− *m*_2_ *r*_1_ sin *α* + *m*_2_/tan *β*]^T^.

According to the kinematic model of the cutting head, the coordinate transformations of C1, C2 and C3 relative to the roadway coordinate system are stored in Table 1 of the Supplementary File Appendix A.

**Table 1:** Orthogonal test table.

"Spherical crown + cylindrical"				"Spherical crown + cone", "spherical crown + cone + cylinder"				
Test level	A (°)	B (mm)	C (mm)	Test level	A (°)	B (mm)	C (mm)	D (°)
1	33	600	390	1	33	600	390	70
2	38	650	400	2	38	650	400	75
3	43	700	410	3	43	700	410	80
4	45	750	420	4	45	750	420	85

O_4_ and O_j_ coordinates of the three cutting heads are the same. The coordinates of O_4_ can be seen from Eq. (2): X_O4_ = *u*_4_ = − (*a*_3_ + *a*_4_ + d) sin *θ*_1_ cos *θ*_2_ − *a*_2_ sin *θ*_1_; Y_O4_ = *v*_4_ = − (*a*_3_ + *a*_4_ + d) cos *θ*_1_ cos *θ*_2_ + *a*_2_ cos *θ*_1_ + *a*_1_ + *a*_0_ + *d*_0_; Z_O4_ = *w*_4_ = (*a*_3_ + *a*_4_ + d) sin *θ*_2_ + *b*_2_ + *b*_1_ + *b*_3_.

The coordinates of O_j_ point are also obtained: X_Oj_ = − *r*_1_*u*_2_ − X_O4_; Y_Oj_ = − *r*_1_*v*_2_ − Y_O4_; Z_Oj_ = − *r*_1_*w*_2_ − Z_O4_.

**Figure 4:**
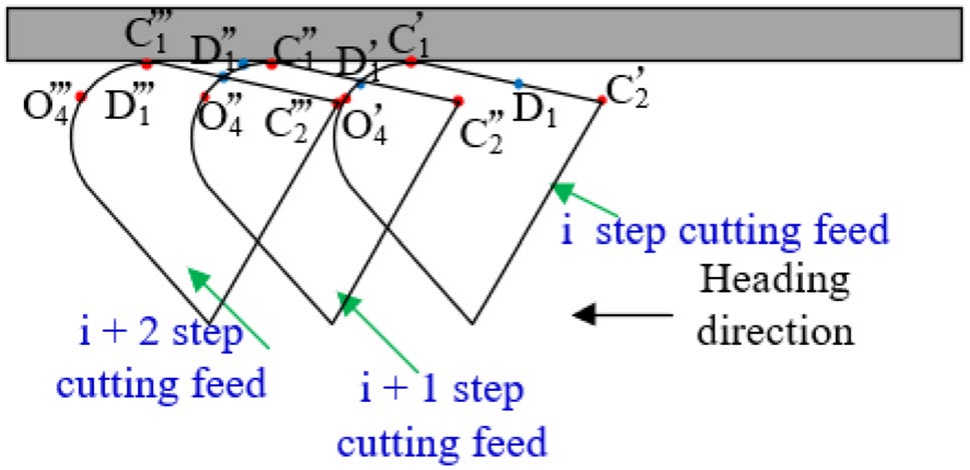
Cutting head cutting roadway forming profile.

As the cutting head is a revolving body, this paper considers that the outer contour formed by cutting coal and rock by the cutting head is determined by the arc O_4_C_1_, line segment C_1_C_2_ and line segment C_2_C_3_ on the section of the cutting head, as shown in Fig. 4.

The outer contour equation of "spherical cap + cylinder" and "spherical cap + conical" cutting head is as follows:

3 $${\left \{ \begin{array}{*{20}c} {{\text{O}}_{4} {\text{C}}_{1} :Z = Z_{{{\text{Oj}}}} + r_{1} \sin \left( {{\text{arccos}}\frac{{Y - Y_{{{\text{Oj}}}} }}{{r_{1} }}} \right)} \\ {Y_{{{\text{Oj}}}} + r_{1} \cos \theta _{2} \le Y \le Y_{{{\text{Oj}}}} + r_{1} \cos \left( {\theta _{2} + \alpha } \right)} \\ {{\text{C}}_{1} {\text{C}}_{2} :Z = \frac{{Z_{{{\text{C}}2}} - Z_{{{\text{C1}}}} }}{{Y_{{{\text{C}}2}} - Y_{{{\text{C1}}}} }}\left( {Y - Y_{{{\text{C2}}}} } \right) + Z_{{{\text{C1}}}} } \\ {Y_{{{\text{C1}}}} \le Y \le Y_{{{\text{C2}}}} } \\ \end{array} \right. }$$

The outer contour equation of the "spherical crown + cone + cylinder" cutting head is as follows:

4 $${\left \{ \begin{array}{*{20}c} {{\text{O}}_{4} {\text{C}}_{1} :Z = Z_{{{\text{Oj}}}} + r_{1} \sin \left( {{\text{arccos}}\frac{{Y - Y_{{{\text{Oj}}}} }}{{r_{1} }}} \right)} \\ {Y_{{{\text{Oj}}}} + r_{1} \cos \theta _{2} \le Y \le Y_{{{\text{Oj}}}} + r_{1} \cos \left( {\theta _{2} + \alpha } \right)} \\ {{\text{C}}_{1} {\text{C}}_{2} :Z = \frac{{Z_{{{\text{C}}2}} - Z_{{{\text{C1}}}} }}{{Y_{{{\text{C}}2}} - Y_{{{\text{C1}}}} }}\left( {Y - Y_{{{\text{C2}}}} } \right) + Z_{{{\text{C1}}}} } \\ {Y_{{{\text{C1}}}} \le Y \le Y_{{{\text{C2}}}} } \\ {{\text{C}}_{2} {\text{C}}_{3} :Z = \frac{{Z_{{{\text{C}}3}} - Z_{{{\text{C2}}}} }}{{Y_{{{\text{C}}3}} - Y_{{{\text{C2}}}} }}\left( {Y - X_{{{\text{C3}}}} } \right) + Z_{{{\text{C2}}}} } \\ {Y_{{{\text{C2}}}} \le Y \le Y_{{{\text{C3}}}} } \\ \end{array} \right. }$$

In the process of tunneling machine, the feeding amount of tunneling is generally 500 mm. The contour line of cutting head obtained by two cutting feeds will intersect D_1_ at the intersection of arc O_4_C_1_ and line segment C_1_C_2_ on the cutting head given by the previous cutting. The intersection can be obtained by solving the equation of Eq. (3).

At this time, the combination of multiple line segments and arcs $\cdots D_{1}^{\prime } C_{1}^{\prime } D_{1}^{\prime \prime } C_{1}^{\prime \prime } D_{1}^{\prime \prime } C_{1}^{\prime \prime \prime } \cdots$ is the roadway surface contour cut by the cutting head, the outer contour formed by coal and rock can be described as

5 $$\left\{ {\begin{array}{*{20}c} \vdots \\ {C_{1}^{\prime } D_{1}^{\prime } :Z = Z_{{O^{\prime } j}} + r_{1} \sin \left( {\arccos \frac{{Y - Y_{{O^{\prime } j}} }}{{r_{1} }}} \right)} \\ {Y_{{C^{\prime } }} \le Y \le Y_{{D_{1}^{\prime } }} } \\ {D_{1}^{\prime } C_{1}^{\prime \prime } :Z = \frac{{Z_{{C^{\prime \prime } }} - Z_{{D_{1}^{\prime } }} }}{{Y_{{C^{\prime \prime } }} - Y_{{D_{1}^{\prime } }} }}\left( {Y - Y_{{C^{\prime \prime } }} } \right) + Z_{{D_{1}^{\prime } }} } \\ {Y_{{D_{1}^{\prime } }} \le Y \le Y_{{C^{\prime \prime } }} } \\ {C_{1}^{\prime \prime } D_{1}^{\prime \prime } :Z = Z_{{O^{\prime \prime } j}} + r_{1} \sin \left( {\arccos \frac{{Y - Y_{{O^{\prime \prime } j}} }}{{r_{1} }}} \right)} \\ {Y_{{C^{\prime \prime } }} \le Y \le Y_{{D_{1}^{\prime \prime } }} } \\ {D_{1}^{\prime \prime } C_{1}^{\prime \prime \prime } :Z = \frac{{Z_{{C_{1}^{\prime \prime \prime } }} - Z_{{D_{1}^{\prime \prime } }} }}{{Y_{{C_{1}^{\prime \prime \prime } }} - Y_{{D_{1}^{\prime \prime } }} }}\left( {Y - Y_{{C^{\prime \prime \prime } }} } \right) + Z_{{D_{1}^{\prime \prime } }} } \\ {Y_{{D_{1}^{\prime \prime } }} \le Y \le Y_{{C_{1}^{\prime \prime \prime } }} } \\ \vdots \\ \end{array} } \right.$$

So far, the mathematical description of the outer contour formed by the cutting head has been completed. The following paper will use MATLAB numerical calculation software to build the simulation program of the outer contour formed by coal and rock, and analyze the influence of cutting feed, cutting Angle, cutting head shape, cutting head radius, cutting head cone Angle and other parameters on the cutting surface morphology characteristics through numerical calculation.

## Simulation analysis of roadway surface topography

### Simulation process

In this paper, EBZ200 roadheader commonly used in coal mine is taken as the research object, and the simulation program of outer contour formed by coal and rock is constructed in MATLAB numerical calculation software, and the parameters in the simulation program are set according to the structure size of EBZ200 roadheader: a0 = 0 mm, a1 = 3000 mm, a2 = 700 mm, a3 = 2470 mm, a4 = 1775 mm, b1 = 1000 mm, b2 = 519 m, b3 = 87 mm, d = 0 mm。r1 = 416 mm, α = 90°, β = 83.5, m1 = 640 mm, m2 = 200 mm.

**Figure 5:**
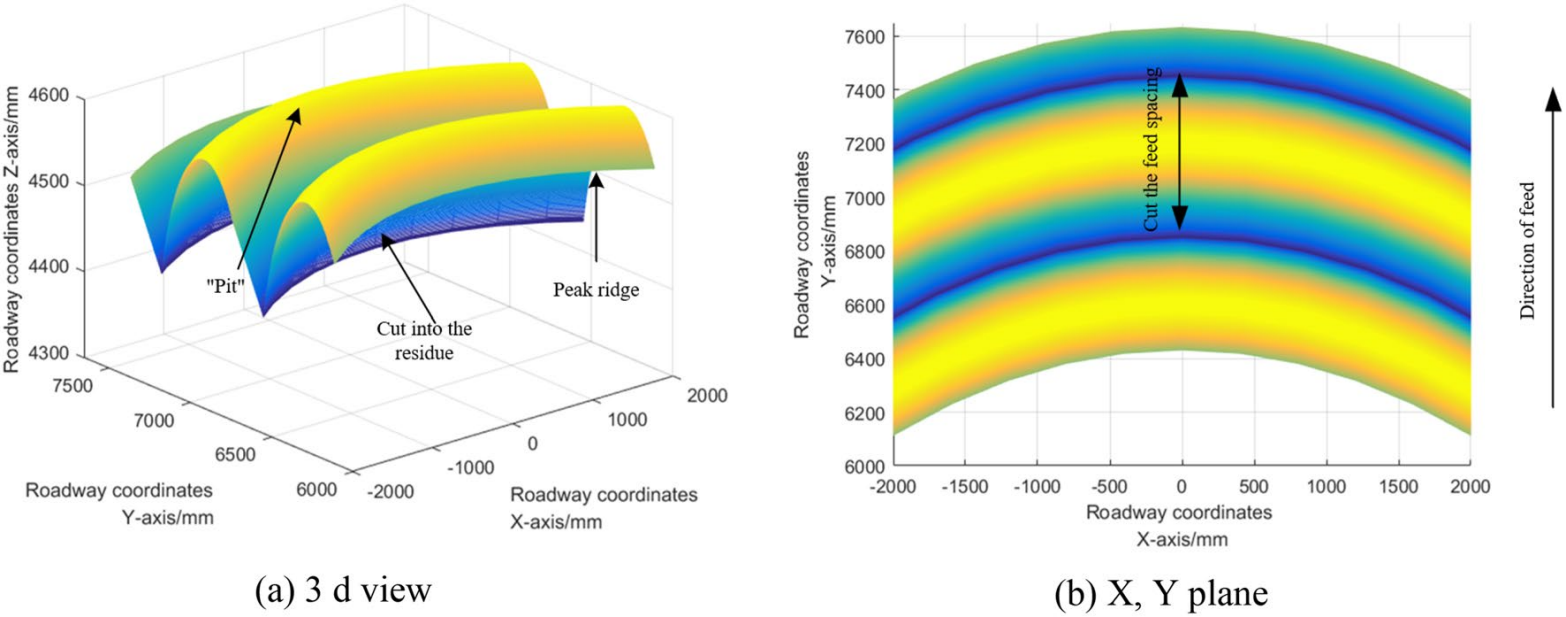
Simulation results of roadway surface morphology.

The cutting feed d0 was set as 550 mm, and the cutting head shapes were respectively selected as "ball crown + cylinder" type, "ball crown + cone" type and "ball crown + cone + cylinder" type. The cutting lifting Angle was 43°. The simulated surface topography of roadway is shown in Fig. 5.

Because the roadway surface topography obtained by the three types of cutting heads is basically similar, and no difference can be seen in the three-dimensional images, only the simulation results of "spherical crown + cone" cutting heads are presented. Details of the differences will be discussed later. It can be seen that after each cutting, "pits" will be formed on the coal rock, and "peaks and ridges" will rise between adjacent "pits". The simulation results are consistent with the expected results shown in Fig. 4.

### Evaluation method of roadway surface topography characteristics

In this paper, the definition of surface roughness Ra of mechanical parts is used to describe the surface topography characteristics of roadway, and the calculation formula is shown in Eq. (6). According to Eq. (6), the default unit of Ra in this paper is mm, without special circumstances, it will not be explained otherwise.

6 $$Ra{ = }\frac{1}{n}\sum\limits_{i = 1}^{n} {\left| {Z_{i} { - }Z_{0} } \right|}$$

Type: *Z*_*0*_ is the required height of roadway; *Z*_i_ is each sampling point, Roadway contour value; n is the sampling number.

### Different cutting head types

**Figure 6:**
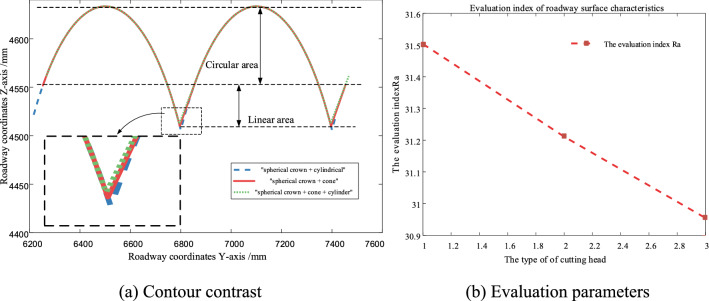
Comparison of roadway section under different cutting head types.

The comparison of the cutting contour of the three cutting heads is shown in Fig. 6a, and the evaluation parameters of roadway surface topography characteristics obtained by the three cutting heads are shown in Fig. 6b.

Under the same parameters, the outer contour curves of three cutting heads can be seen that the outer contour of cutting heads is composed of straight line and circular arc segment, the straight line segment is obtained by the action of the cylinder or cone of the cutting head and coal rock, and the circular arc segment is obtained by the interaction of the spherical crown of the cutting head and coal rock.

According to the evaluation index of roadway topography characteristics, it can be seen that different types of cutting heads form different roughness profiles on coal rock after cutting: the "spherical crown + cylindrical" cutting head has the largest roughness of the outer contour formed on coal rock, the "spherical crown + cone" cutting head is the second, and the "spherical crown + cone + cylindrical" cutting head is the smallest. It indicates that the roadway surface obtained by "spherical crown + cylinder" cutting head is the most uneven, while the roadway surface obtained by "spherical crown + cone + cylinder" cutting head is the most flat. Therefore, the suggestion given by this part of the research is that when selecting the cutting head, more attention should be paid to the "spherical crown + cone + cylinder" cutting head.

## Experimental study

**Figure 7:**
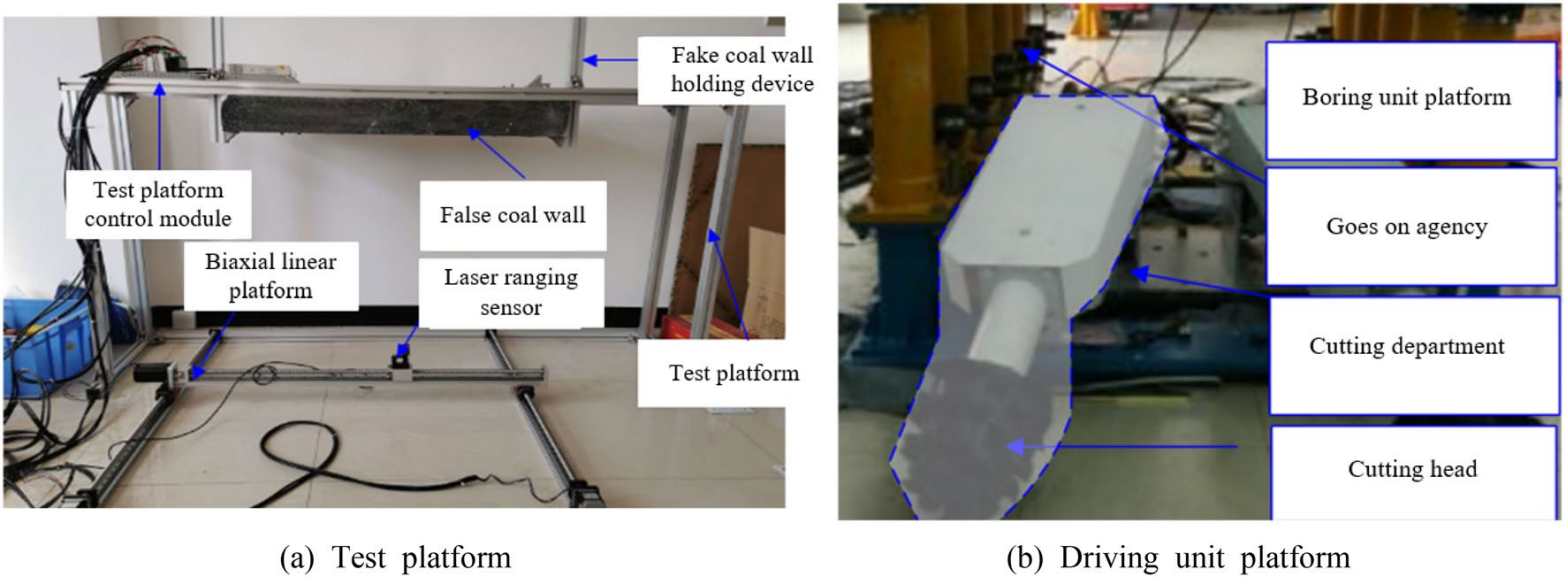
Experimental platform.

Below, the self-made experimental platform is used for experimental research, which consists of two parts: the test platform and the boring unit platform, as shown in Fig. 7.

The self-made test platform is mainly composed of frame, false coal wall, biaxial linear slide platform and control module. The platform of the tunneling unit is a model prototype of the tunneling unit made by ourselves, and the parameters of all materials are obtained by similar proportion conversion according to the parameters of EBZ200 tunneling machine. In this paper, the influence of cutting head geometric factors on roadway surface topography characteristics was mainly studied. In order to avoid the influence of cutting load and cutting vibration on the research results, high-density hard foam was used as the fake coal wall.

**Figure 8:**
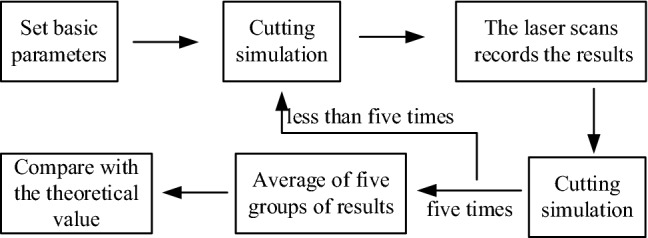
The experimental process.

**Figure 9:**
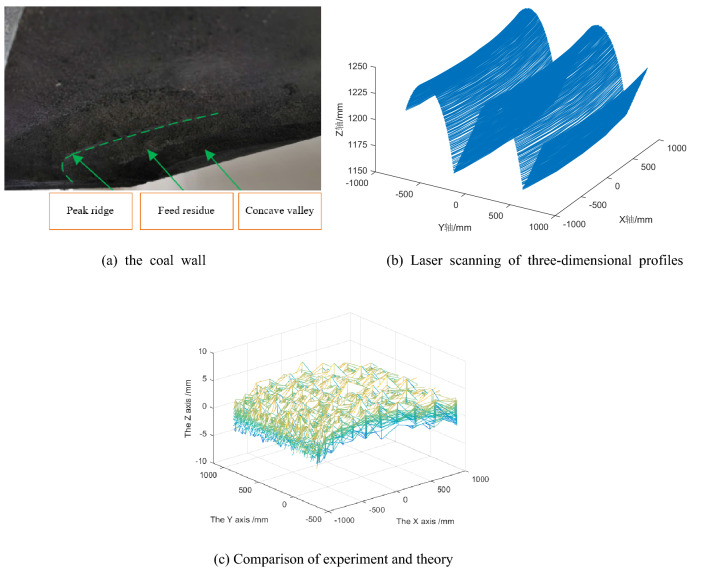
Experimental results of cutting false coal wall.

The "spherical crown + cone + cylinder" type cutting head was used in this experiment, with a cone Angle of 75° and a length of 910 mm. The cutting Angle is 43° and the feed step is 660 mm. The experimental process is shown in Fig. 8. The biaxial linear slide platform of the test platform and the high-precision laser displacement sensor mounted on it were used to scan the fake coal wall after cutting, and the 3D contour was obtained and its comparison with theoretical research results was shown in Fig. 9.

The experimental results show that the error of theoretical calculation and experimental test is mainly concentrated within ± 2 mm, mainly due to the particle size of the foam used to make the fake coal wall. The error of some measuring points is ± 6 mm, mainly because the foam is torn and dropped under the action of the pick. In general, the feasibility of the theoretical research method and the correctness of the results can be verified through experiments.

## Multivariate regression fitting

The orthogonal test method was used to study the roadway surface topography characteristics formed by three cutting heads under different cutting angles, cutting feed, cutting head crown radius and cutting head conical Angle. In order to facilitate the study, the test factors of cutting lifting Angle, cutting feed, cutting head crown radius and cutting head conical Angle are respectively A, B, C and D. Considering that the "spherical crown + cylindrical" cutting head has no conical Angle, the orthogonal experimental table is designed separately. See Table 1 for specific parameters.

According to the above data, L_16_(4^3^) orthogonal test with 4 levels and 3 factors was designed for "spherical crown + cylinder" cutter head, and L_16_(4^4^) orthogonal test with 4 levels and 4 factors was designed for "spherical crown + cone" cutter head and "spherical crown + cone + cylinder" cutter head. Through theoretical solution, the orthogonal test results are stored in Table 2 of the Supplementary File Appendix A.

**Table 2:** Validation indicators.

	"Spherical crown + cylinder" type	"Spherical crown + conical" type	"Spherical crown + conical + cylindrical" type
ESS	0.17185	0.14598	0.01938
TSS	16,522	13,132	13,580
R2	0.99999	0.99999	0.9999985

Based on orthogonal test results, second order linear regression was performed on the functional relationship between each factor and evaluation parameters, and the functional relationship between each influencing factor and evaluation parameters was obtained as follows:

8 $$\begin{gathered} Ra_{1} = \beta_{0} + \beta_{1} A + \beta_{2} B + \beta_{3} C + \beta_{4} D + \beta_{5} A^{2} \\ + \beta_{6} AB + \beta_{7} AC + \beta_{8} B^{2} + \beta_{9} BC + \beta_{10} C^{2} \\ \end{gathered}$$

9 $$\begin{gathered} Ra_{2} = \beta_{0} + \beta_{1} A + \beta_{2} B + \beta_{3} C + \beta_{4} D + \beta_{5} A^{2} + \beta_{6} AB + \beta_{7} AC + \\ \beta_{8} AD + \beta_{9} B^{2} + \beta_{10} BC + \beta_{11} BD + \beta_{12} C^{2} + \beta_{13} CD + \beta_{14} D^{2} \\ \end{gathered}$$

10 $$\begin{gathered} Ra_{3} = \beta_{0} + \beta_{1} A + \beta_{2} B + \beta_{3} C + \beta_{4} D + \beta_{5} A^{2} + \beta_{6} AB + \beta_{7} AC + \hfill \\ \beta_{8} AD + \beta_{9} B^{2} + \beta_{10} BC + \beta_{11} BD + \beta_{12} C^{2} + \beta_{13} CD + \beta_{14} D^{2} \hfill \\ \end{gathered}$$

Ra_1_, Ra_2_ and Ra_3_ represent the evaluation parameters of the influence of "spherical crown + cylindrical" cutting head, "spherical crown + conical" cutting head and "spherical crown + conical + cylindrical" cutting head on the surface topography characteristics of roadway. The detailed values of the above coefficients are listed in Table 3 of the Supplementary Document Appendix A.

**Table 3:** Basic parameter setting.

"Spherical crown + conical + cylindrical" type			
A (°)	(33, 45)	C (mm)	400
B (mm)	(600, 750)	D (°)	80

After obtaining the second-order linear regression equation between each factor and the evaluation parameters, the accuracy and rationality of the model should be analyzed. This paper focuses on R detection to verify the significance of the regression equation. The calculation formula of significance evaluation parameters is as follows^22^:

11 $$R^{2} = 1 - \frac{ESS}{{TSS}}$$

where: ESS is the sum of squares of residuals and TSS is the sum of squares of deviations.

The calculated sum of squares of deviations, sum of squares of residuals and R^2^ values of the three groups are shown in Table 5. The sum of squared deviations can be regarded as the sum of the sum of squared residuals and the regression error. When the residual error is smaller, the fitted equation is more significant, that is, when R2 is closer to 1, the fitted equation is more significant. According to the analysis in Table 2, the regression equations corresponding to the three cutting heads have a very high degree of fit.

## The surface topography characteristics of roadway were studied by regression equation

The reliability of the three regression equations is simply verified by R detection above. After the regression equation is obtained, the regression fitting equation can be used to predict the characteristic evaluation parameters of roadway surface topography. Compared with the theoretical calculation deduced in “Kinematics model of cutting head”, the complexity can be simplified. Under the premise of given several factors, the surface topography characteristics of roadway under the joint action of two different factors are studied. At the same time, matlab was used to compare the fitted regression calculated value with the theoretical calculated value derived above to further test the reliability of the regression fitting equation.

The theoretical analysis shows that the roadway surface characteristics formed by the three cutting heads have the same changing trend under the influence of various factors. Therefore, only the results of "spherical crown + cone + cylinder" type cutting head are shown in the main text, and the rest are stored in the Supplementary Document.

### Different cutting lifting angles and cutting feed

In order to facilitate the study of the influence of different cutting lifting angles and cutting feed on the evaluation parameters of roadway surface topography characteristics. Make some Settings for each influencing factor, and the specific values are shown in Table 3.

**Figure 10:**
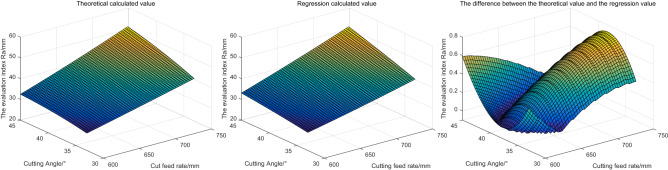
The influence of different cutting angles and cutting feed rate on the evaluation index.

Based on the above parameter Settings, the evaluation index is solved and shown in Fig. 10. In Fig. 10, from left to right are respectively the theoretical calculation value, regression calculation value and the difference between the evaluation parameters obtained under the two calculation methods (theoretical value minus regression calculation value, the same below).

According to Fig. 10, the following conclusions can be drawn: with the increase of cutting Angle and cutting feed, the roughness of the outer profile formed by different cutting heads after cutting increases. It indicates that with the increase of cutting lifting Angle and cutting feed, the roadway smoothness obtained by cutting decreases. The larger the cutting Angle and feed, the more need to accurately control the flatness of roadway. The calculated values of regression and theoretical calculation are generally consistent, and the maximum difference between them is not more than 0.8 mm. The trend of the regression value is consistent with the theoretical value.

### Different cutting Angle and radius of cutting head crown

In order to facilitate the study of the influence of different cutting lifting angles and the radius of cutting head on the evaluation parameters of roadway surface topography characteristics. Make some Settings for each influencing factor, and the specific values are shown in Table 4.

**Table 4:** Basic parameter setting.

"Spherical crown + conical + cylindrical" type			
A (°)	(33, 45)	C (mm)	(390, 420)
B (mm)	700	D (°)	80

**Figure 11:**
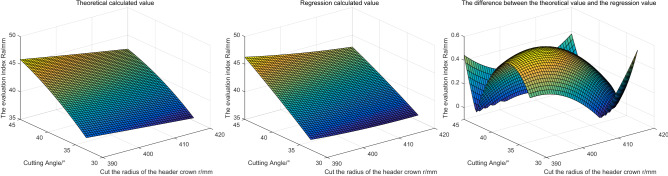
The influence of different cutting angles and the radius of cutting head crown on the evaluation index.

Based on the above parameter Settings, the evaluation indexes obtained by the two methods are shown in Fig. 11.

Based on Fig. 11, it can be seen that: with the increase of the cutting Angle, the roughness of the contour formed by the cutting head on the coal rock increases. With the increase of the radius of the cutting head, the roughness of the outer profile on the coal rock gradually decreases. It indicates that the smaller the cutting lifting Angle is, the larger the radius of the spherical crown is and the smoother the roadway surface is formed.

### Different cutting lifting Angle and cutting head conical Angle

In order to study the evaluation index affected only by cutting lifting Angle and cutting head conical Angle, make some Settings for each influencing factor, and the specific values are shown in Table 5.

**Table 5:** Basic parameter setting.

"Spherical crown + conical + cylindrical" type			
A (°)	(33, 45)	C (mm)	400
B (mm)	700	D (°)	(70, 85)

**Figure 12:**
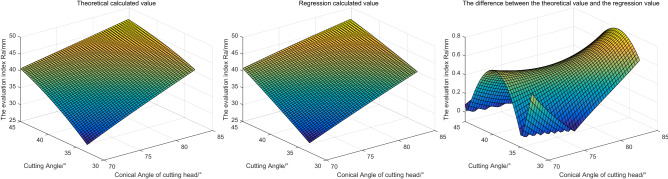
The influence of different cutting Angle and conical Angle of cutting head on evaluation index.

Based on the above parameter Settings, the evaluation indexes obtained by the two methods are shown in Fig. 12.

Based on Fig. 12, we can see that: with the increase of cutting Angle and cutting feed, the roughness of the outer profile on coal rock increases. This indicates that in practice, the cutting Angle and the cutting step should be selected as small as possible. In addition, the regression calculated values are very close to the theoretical calculated values, and the maximum error is not more than 0.8 mm. The trend of the regression value is consistent with the theoretical value.

### Different cutting feed and cutting crown radius

In order to study the evaluation indexes of roadway surface topography characteristics affected only by cutting feed and the radius of cutting head cap, make some Settings for each influencing factor, and the specific values are shown in Table 6.

**Table 6:** Basic parameter setting.

"Spherical crown + conical + cylindrical" type			
A (°)	40	C (mm)	(390, 420)
B (mm)	(600, 750)	D (°)	80

**Figure 13:**
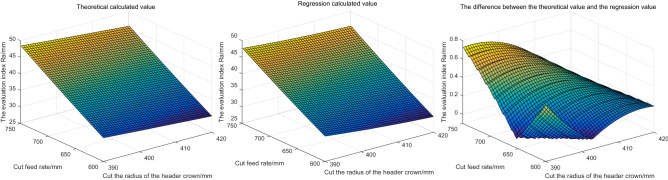
The influence of different cut feed rate and cut head crown radius on the evaluation index.

The evaluation index results obtained through theoretical calculation and regression calculation are shown in Fig. 13.

Based on Fig. 13, it can be seen that: the increase of cutting feed and the decrease of cutting head crown radius will lead to the larger evaluation index obtained by cutting and the rougher roadway surface profile. The maximum difference between the calculated value of regression equation and the calculated value of theory is not more than 0.8 mm. The trend of the regression value is consistent with the theoretical value.

### Different cutting feed and cutting head conical Angle

In order to study the evaluation index of roadway surface topography characteristics under the common influence of cutting feed and conical Angle of cutting head, make some Settings for each influencing factor, and the specific values are shown in Table 7.

**Table 7:** Basic parameter setting.

"Spherical crown + conical + cylindrical" type			
A (°)	40	C (mm)	400
B (mm)	(600, 750)	D (°)	(170, 85)

**Figure 14:**
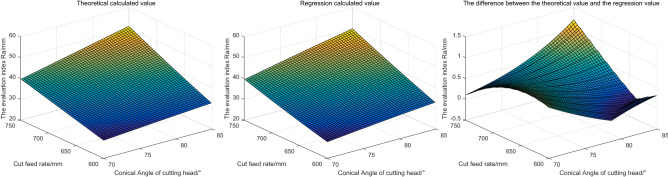
The influence of different cutting feed rate and conical Angle of cutting head on evaluation index.

The characteristic evaluation index results obtained through theoretical calculation and regression calculation are shown in Fig. 14.

Based on Fig. 14, it can be seen that: under the current setting, the evaluation index increases with the increase of cutting feed and the taper Angle of cutting head. This indicates that the roadway surface is rougher when the feed rate and cone Angle are larger. The regression fitting equations can accurately predict the evaluation index only when cutting feed rate and cutting head cone Angle change, and the maximum error is less than 1.5 mm. The trend of the regression value is consistent with the theoretical value.

### Different cutting head crown radius and cutting head conical Angle

In order to study the evaluation index of roadway surface topography characteristics under the joint influence of the radius of the cutting head and the conical Angle of the cutting head. Make some Settings for each influencing factor, and the specific values are shown in Table 8.

**Table 8:** Basic parameter setting.

"Spherical crown + conical + cylindrical" type			
A (°)	40	C (mm)	(390, 420)
B (mm)	700	D (°)	(70, 85)

**Figure 15:**
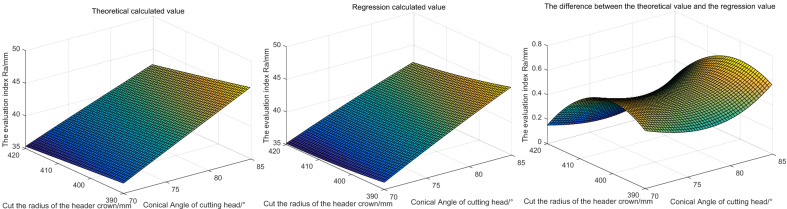
The influence of different cut head crown radius and cut head conical Angle on the evaluation index.

The evaluation index results obtained through theoretical calculation and regression calculation are shown in Fig. 15.

Based on Fig. 15, we can see that: under the current setting, the reduction of the radius of the cutting head crown and the increase of the conical Angle of the cutting head will lead to the increase of the evaluation index. The larger the evaluation index, the rougher the roadway profile. The regression equation can reliably predict the evaluation index when only the radius of the cutting head crown and the conical Angle of the cutting head change, and the maximum error is not more than 0.8 mm. The trend of the regression value is consistent with the theoretical value.

Above, six groups of roadway surface topography characteristics evaluation indexes under the joint action of two different factors were analyzed respectively, and the simulation results of each group were in line with expectations. Based on the above 6 groups of simulation results, the following conclusions can be drawn.

1. The three regression fitting equations can be used to study the influence of single factor on roadway surface topography characteristics, and the results are consistent with theoretical analysis.
2. Several different influencing factors are combined in pairs, and the simulation results can correctly reflect the relationship between a single factor and the evaluation index of roadway surface topography characteristics, with high computational accuracy, and the overall change trend is consistent with the theoretical analysis. It shows that the three regression fitting equations can accurately calculate the evaluation index within a certain range. That is, given any set of factors within a certain range, the three regression fitting equations can be solved instead of theoretical solution, which can play a simplification effect.

## Conclusion

At present, more scholars study roadway forming from the perspective of roadheader cutting arm control. Based on the forming mechanism of roadway, this paper studies the influence of cutting head type, cutting head geometric size and feed rate of roadheader on roadway profile. Compared with the simplification of cutting head by other scholars, it has more guiding significance for practical production.

This paper mainly studies the modeling method of cutting head movement trajectory, the mathematical model of cutting head cutting roadway contour and the simulation method of roadway contour. The formation mechanism of roadway contour obtained by cutting roadway with cutting head is studied. Then, from the perspective of geometric motion, the influence law of various factors on the roadway topography characteristics is analyzed.

1. According to the surface formation mechanism of cutting roadway, cutting feed, cutting Angle, cutting head shape, cutting head radius, cutting head cone Angle and other parameters have an impact on the surface morphology characteristics of cutting.
2. "Spherical cap + cylinder" type cutting head cut the roadway surface is the most uneven, "spherical cap + cone + cylinder" type cutting head cut the roadway surface is the most smooth. Therefore, in the selection of cutting head should be considered more "ball cap + cone + cylinder" type cutting head.
3. With the decrease of cutting lifting Angle, cutting feed, cutting head cone Angle and the increase of cutting head crown radius, the roadway smoothness obtained by cutting is higher. Therefore, choosing the cutting head with larger radius of spherical crown and smaller cone Angle of cutting head is beneficial to improve the smoothness of roadway. Cutting feed should not be selected too large, the selection of feed should take into account the tunneling efficiency and the smoothness of roadway forming. The higher the cutting Angle is, the more accurate control of roadway flatness is needed.
4. The orthogonal test was designed, and the fitting regression equation of each influencing factor and evaluation index Ra was obtained by combining the orthogonal test results. The regression equation is used to predict the relationship between each influencing factor and the evaluation index, and the prediction conclusion is consistent with the theoretical analysis.

## Supplementary Information

Supplementary Information.

## Data Availability

All data generated or analysed during this study are included in this published article [and its supplementary information files].
