# Supplementary material for: Modeling and characteristic analysis of roadway profile under the influence of multiple factors
Source: Sci Rep. 2022 Nov 18;12:19879. doi: 10.1038/s41598-022-24205-6 (PMC9674606; doi:10.1038/s41598-022-24205-6)
Supplement: Supplementary file 1 — Supplementary Information. [file 41598_2022_24205_MOESM1_ESM.pdf]

Modeling and characteristic analysis of roadway profile under the influence of multiple factors

LIU Zhixiang[1]， ZOU Kang*[1]， XIE Miao[1]，XIE Chunxue[2]，SUN Zhan[1]

(1. Research Institute of mineral resources development and utilization technology and equipment, Liaoning University of engineering and technology, Liaoning Province, 123000; 2. School of mechanics and engineering, Liaoning University of engineering and technology, Liaoning Province, 123000)

Appendix A Original experimental data

Table 1 coordinate representation of key nodes of three cutting heads

cutting head	Coordinates of point C_1			Coordinates of point C_2			Coordinates of point C_3		
	X_{c1}	Y_{c1}	Z_{c1}	X_{c2}	Y_{c2}	Z_{c2}	X_{c3}	Y_{c3}	Z_{c3}
"Spherical crown + cylinder" type	$-r_1(1-\cos\alpha)u_2+u_3r_1\sin\alpha+u_4$	$-r_1(1-\cos\alpha)v_2+v_3r_1\sin\alpha+v_4$	$-r_1(1-\cos\alpha)w_2+w_3r_1\sin\alpha+w_4$	$-(r_1+m_1)u_2+u_3r_1\sin\alpha+u_4$	$-(r_1+m_1)v_2+v_3r_1\sin\alpha+v_4$	$-(r_1+m_1)w_2+w_3r_1\sin\alpha+w_4$	-	-	-
"spherical crown + conical"	$-r_1(1-\cos\alpha)u_2+u_3r_1\sin\alpha+u_4$	$-r_1(1-\cos\alpha)v_2+v_3r_1\sin\alpha+v_4$	$-r_1(1-\cos\alpha)w_2+w_3r_1\sin\alpha+w_4$	$-(r_1+m_1)u_2+(r_1\sin\alpha+(m_1+r_1\cos\alpha)/\tan\beta)u_3+u_4$	$-(r_1+m_1)v_2+(r_1\sin\alpha+(m_1+r_1\cos\alpha)/\tan\beta)v_3+v_4$	$-(r_1+m_1)w_2+(r_1\sin\alpha+(m_1+r_1\cos\alpha)/\tan\beta)w_3+w_4$	-	-	-
"spherical crown + conical + cylindrical"	$-r_1(1-\cos\alpha)u_2+u_3r_1\sin\alpha+u_4$	$-r_1(1-\cos\alpha)v_2+v_3r_1\sin\alpha+v_4$	$-r_1(1-\cos\alpha)w_2+w_3r_1\sin\alpha+w_4$	$-(r_1+m_1)u_2+(r_1\sin\alpha+(m_1+r_1\cos\alpha)/\tan\beta)u_3+u_4$	$-(r_1+m_1)v_2+(r_1\sin\alpha+(m_1+r_1\cos\alpha)/\tan\beta)v_3+v_4$	$-(r_1+m_1)w_2+(r_1\sin\alpha+(m_1+r_1\cos\alpha)/\tan\beta)w_3+w_4$	$-(r_1+m_1+m_2)u_2+(r_1\sin\alpha+m_1/\tan\beta)u_3+u_4$	$-(r_1+m_1+m_2)v_2+(r_1\sin\alpha+m_1/\tan\beta)v_3+v_4$	$-(r_1+m_1+m_2)w_2+(r_1\sin\alpha+m_1/\tan\beta)w_3+w_4$

In table 1, $u_1=\cos\theta_1$； $u_2=-\sin\theta_1\cos\theta_2$； $u_3=-\sin\theta_1\sin\theta_2$； $u_4=-(a_3+a_4+d)\sin\theta_1\cos\theta_2-a_2\sin\theta_1$； $v_1=\sin\theta_1$； $v_2=\cos\theta_1\cos\theta_2$； $v_3=-\cos\theta_1\sin\theta_2$； $v_4=(a_3+a_4+d)\cos\theta_1\cos\theta_2+a_2\cos\theta_1+a_1+a_0+d_0$； $w_1=0$； $w_2=\sin\theta_2$； $w_3=\cos\theta_2$； $w_4=(a_3+a_4+d)\sin\theta_2+b_2+b_1+b_3$.

Table 2 Orthogonal test results

(a) "spherical crown + cylindrical" type cutting head

Test level	A/°	B/mm	C/mm	Ra/mm
1	33	600	390	32.9490265488343
2	33	650	400	37.7440580974549
3	33	700	410	42.6750338250022
4	33	750	420	47.7217761937054
5	38	600	400	32.8380718980857
6	38	650	390	40.2594849973033
7	38	700	420	43.3567851476982
8	38	750	410	51.5795429689533
9	43	600	410	32.0993104303518
10	43	650	420	37.3904508709696
11	43	700	390	48.7773166926029
12	43	750	400	55.2393743467668
13	45	600	420	31.1343178166332
14	45	650	410	38.7050575330459
15	45	700	400	47.6089003968922
16	45	750	390	57.8551396563571
Ⅰ	161.089894664997	129.020726693905	179.840967895098	
Ⅱ	168.033885012040	154.099051498774	173.430404739200	
Ⅲ	173.506452340691	182.418036062196	165.058944757353	
Ⅳ	175.303415402928	212.395833165783	159.603330029006	
K_1	40.2724736662492	32.2551816734762	44.9602419737744	
K_2	42.0084712530101	38.5247628746934	43.3576011847999	
K_3	43.3766130851728	45.6045090155489	41.2647361893383	
K_4	43.8258538507321	53.0989582914456	39.9008325072516	
R	3.55338018448290	20.8437766179694	5.05940946652279	

(b) "spherical crown + conical" type cutting head

Test level	A/°	B/mm	C/mm	D/°	Ra/mm
1	33	600	390	70	23.9820200179432
2	33	650	400	75	29.5353956303584
3	33	700	410	80	36.2617273194527
4	33	750	420	85	44.2949203944608
5	38	600	400	80	30.8641007950628
6	38	650	390	85	38.7391067509939
7	38	700	420	70	34.4585612924810
8	38	750	410	75	41.7907909588647
9	43	600	410	85	31.8927707575704
10	43	650	420	80	36.0883624332266
11	43	700	390	75	42.8132608128061
12	43	750	400	70	44.5698316207894
13	45	600	420	75	30.5883458208580
14	45	650	410	70	35.9469048438466
15	45	700	400	85	46.4936961540947
16	45	750	390	80	52.7683358686191
Ⅰ	134.074063362215	117.327237391434	158.302723450362	138.957317775060	
Ⅱ	145.852559797402	140.309769658425	151.463024200305	144.727793222887	
Ⅲ	155.364225624392	160.027245578835	145.892193879735	155.982526416361	
Ⅳ	165.797282687418	183.423878842734	145.430189941026	161.420494057120	
K_1	33.5185158405537	29.3318093478586	39.5756808625906	34.7393294437650	
K_2	36.4631399493506	35.0774424146064	37.8657560500763	36.1819483057218	
K_3	38.8410564060981	40.0068113947086	36.4730484699336	38.9956316040903	
K_4	41.4493206718546	45.8559697106835	36.3575474852566	40.3551235142800	
R	7.93080483130087	16.5241603628249	3.21813337733398	5.61579407051492	

(c) "spherical crown + cone + cylinder" type cutting head

Test level	A/°	B/mm	C/mm	D/°	Ra/mm
1	33	600	390	70	22.8167380595098
2	33	650	400	75	28.8291599239505
3	33	700	410	80	36.0501215222023
4	33	750	420	85	44.5360106663302
5	38	600	400	80	30.6920439165168
6	38	650	390	85	38.9603192907900
7	38	700	420	70	33.3106706162326
8	38	750	410	75	41.0460073118746
9	43	600	410	85	32.0105029620314
10	43	650	420	80	35.9408010271995
11	43	700	390	75	42.2118746530945
12	43	750	400	70	43.4619355912146
13	45	600	420	75	30.3177437235787
14	45	650	410	70	35.2438634894754
15	45	700	400	85	46.6851681636099
16	45	750	390	80	52.5528436559430
Ⅰ	132.232030171993	115.837028661637	156.541775659337	134.833207756432	
Ⅱ	144.009041135414	138.974143731415	149.668307595292	142.404785612498	
Ⅲ	153.625114233540	158.257834955139	144.350495285584	155.235810121862	
Ⅳ	164.799619032607	181.596797225362	144.105226033341	162.192001082762	
K_1	33.0580075429982	28.9592571654092	39.1354439148343	33.7083019391081	
K_2	36.0022602838535	34.7435359328538	37.4170768988230	35.6011964031246	
K_3	38.4062785583850	39.5644587387848	36.0876238213959	38.8089525304654	
K_4	8.14189721515353	16.4399421409314	3.10913740649904	6.83969833158229	
R	8.14189721515353	16.4399421409314	3.10913740649904	6.83969833158229	

Table 3 Fitting equation coefficients

coefficient	"Spherical crown + cylinder" type	"spherical crown + conical" type	"spherical crown + conical + cylindrical" type
β_0	-11.1705299263402	0	0
β_1	0.506191568089685	4.63109571005070	4.48487924252028
β_2	0.113012697960950	-0.338985554484358	-0.378365025303028
β_3	-0.0990459847352096	-0.352928056441452	-0.419170746672541
β_4	-0.0114190288318290	2.14188538869126	2.80468406683547
β_5	0.00252742952033520	-0.00911695770162027	-0.00543571454660623
β_6	-0.00269178507347562	0.00356404678568583	0.00351564721611680
β_7	0.000122486807467961	-0.00761132330722278	-0.00717438720785583
β_8	-0.000596621971090373	-0.0333119132048305	-0.0367168020006430
β_9	0.000596842767219570	5.22701741137194e-05	5.35165121810571e-05
β_{10}	-	0.000104673225659865	0.000184996505405533
β_{11}	-	0.00235899720709182	0.00243901863342171
β_{12}	-	0.00103731306072831	0.00109385096559956
β_{13}	-	-0.00419176491910143	-0.00484703321699511
β_{14}	-	-0.00187800177426990	-0.00338843229197064

Table 1 is the coordinate transformation formula for C1, C2, and C3 relative to the road coordinate system.
Table 2 shows the results of three orthogonal tests
Table 3 shows the coefficients of the three regression fitting equations

Appendix A Results of multivariate analysis

1 Different cutting lifting angles and cutting feed

Table1 Basic parameter setting

	"Spherical crown + cylinder" type	"spherical crown + conical" type
A/°	(33,45)	(33,45)
B/mm	(600,750)	(600,750)
C/mm	400	400
D/°		80

The results of regression calculation and theoretical calculation are shown in Fig.2.

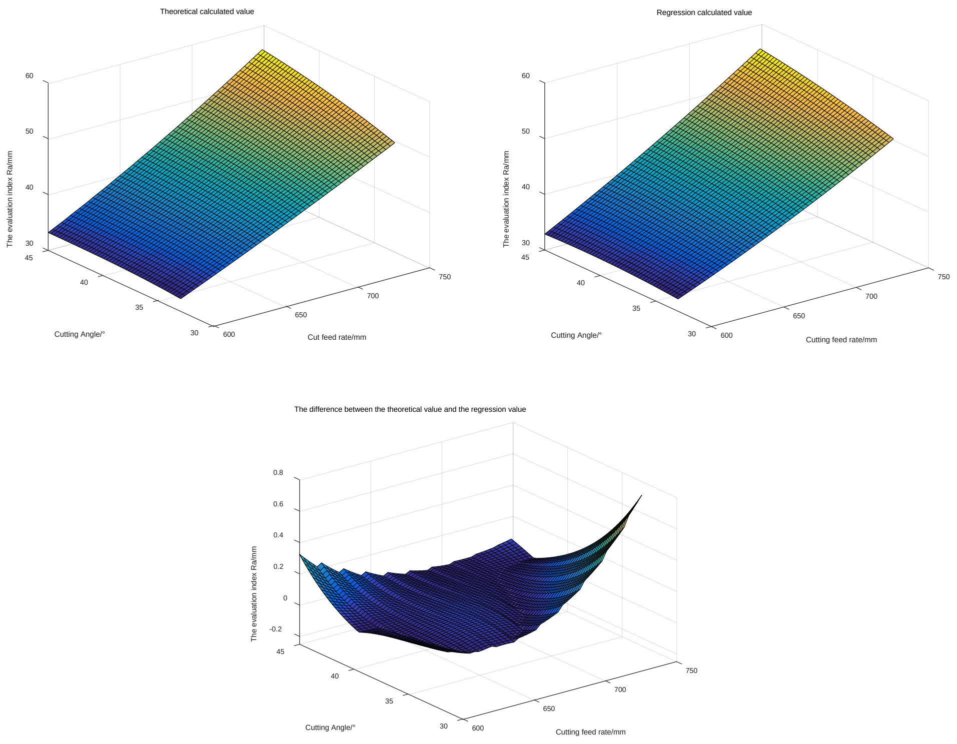

(a) "spherical crown + cylindrical" type cutting head

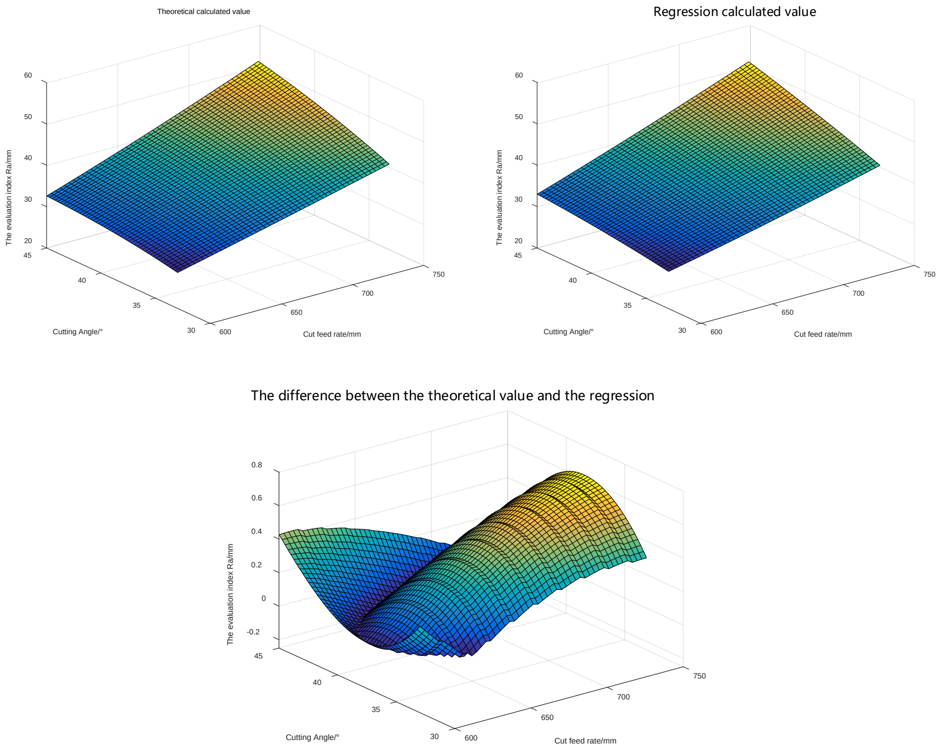

(b) "spherical crown + conical" type cutting head

Fig 1 Different cutting lifting angles and cutting feed

2 Different cutting Angle and radius of cutting head crown

Table 2 Basic parameter setting

	"Spherical crown + cylinder" type	"spherical crown + conical" type
A/°	(33,45)	(33,45)
B/mm	700	700
C/mm	(390，420)	(390，420)
D/°		80

The results of regression calculation and theoretical calculation are shown in Fig.2.

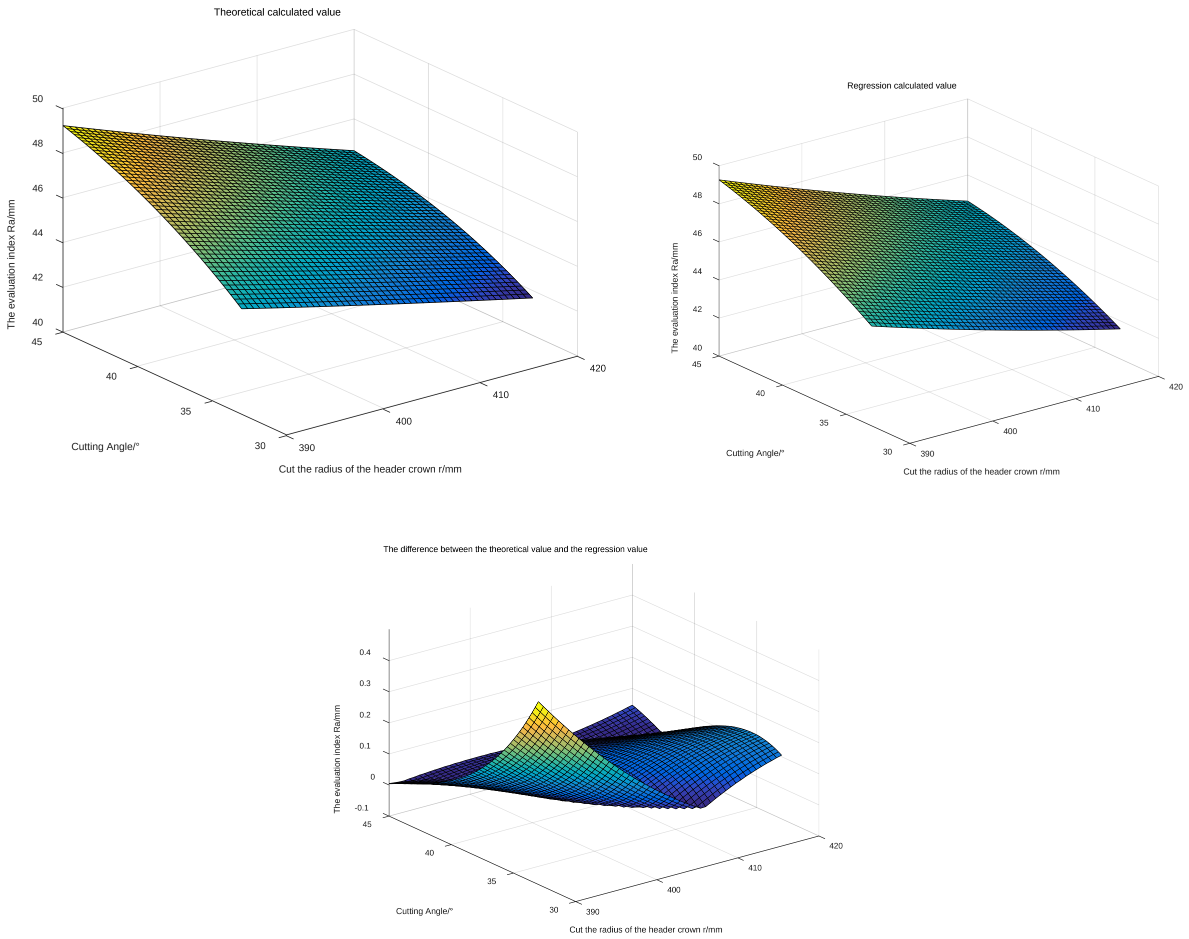

(a) "spherical crown + cylindrical" type cutting head

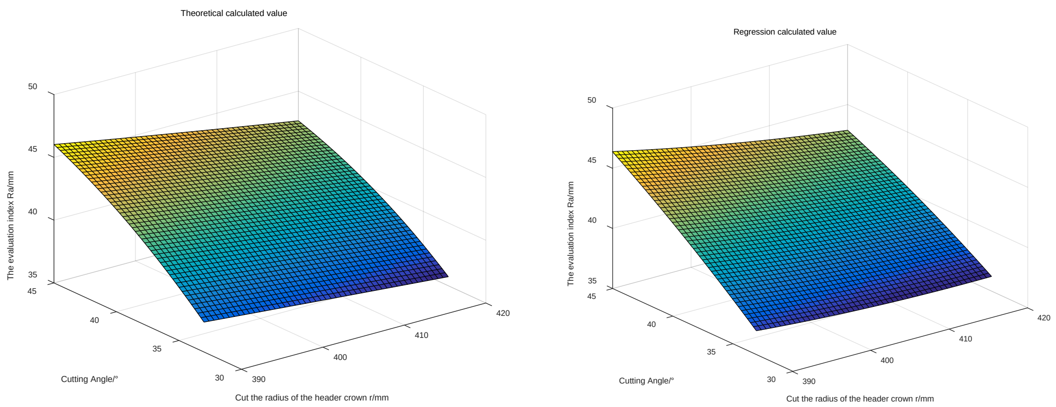

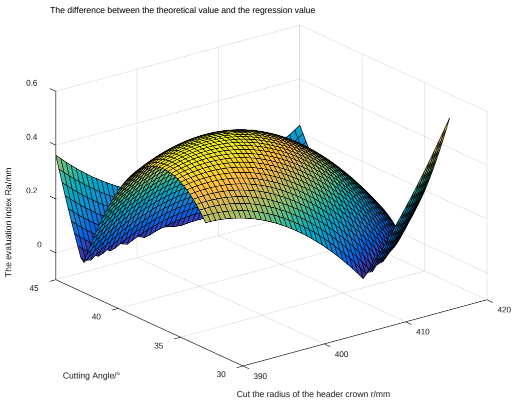

(b) "spherical crown + conical" type cutting head

Fig 2 Different cutting Angle and radius of cutting head crown

3 Different cutting lifting Angle and cutting head conical Angle

Table 3 Basic parameter setting

"spherical crown + conical" type			
A/°	(33,45)	C/mm	400
B/mm	700	D/°	(70，85)

Based on the above parameter Settings, the evaluation indexes obtained by the two methods are shown in Fig.3.

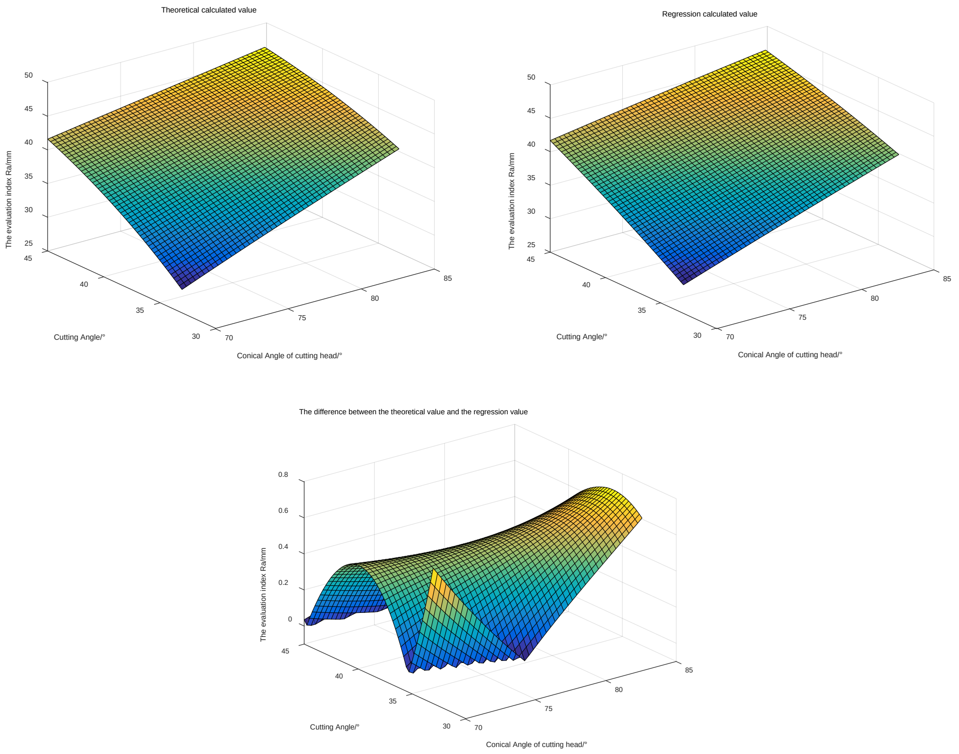

(a) "spherical crown + conical" type cutting head

Fig 3 Different cutting lifting Angle and cutting head conical Angle

4 Different cutting feed and cutting crown radius

Table 4 Basic parameter setting

	"Spherical crown + cylinder" type	"spherical crown + conical" type
A/°	40	40
B/mm	(600，750)	(600，750)
C/mm	(390，420)	(390，420)
D/°		80

The evaluation index results obtained through theoretical calculation and regression calculation are shown in Fig.4.

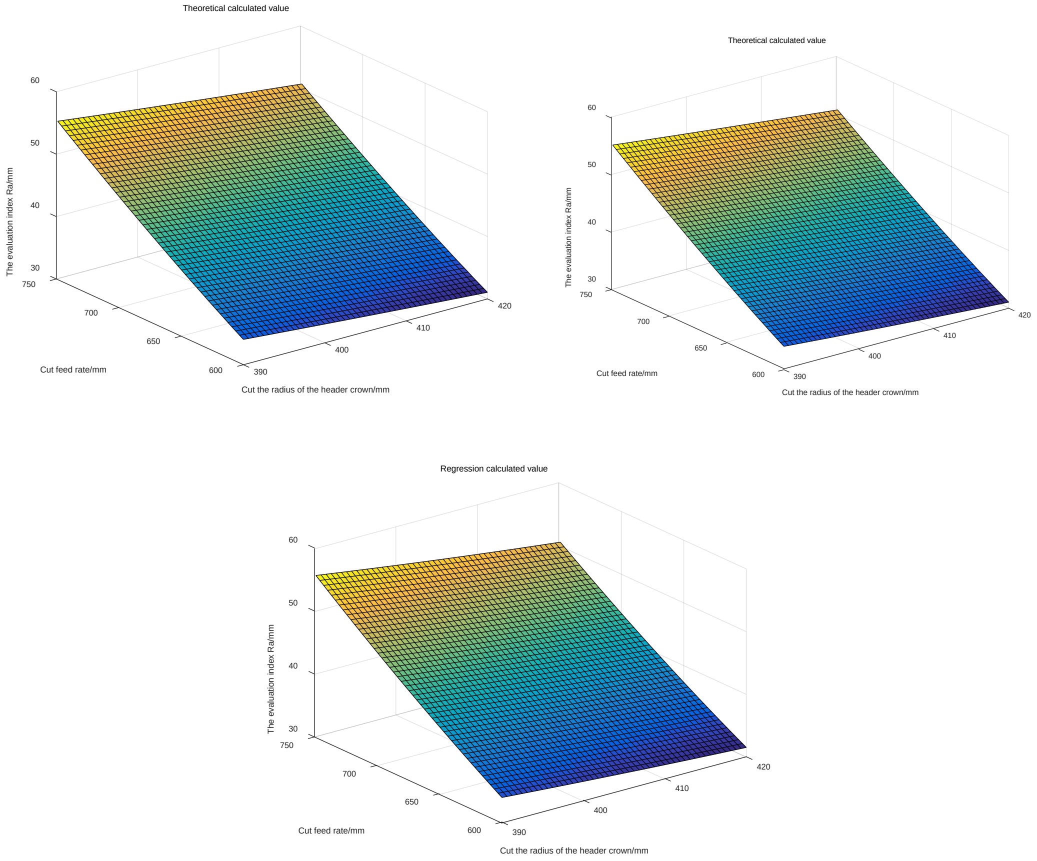

(a) "spherical crown + cylindrical" type cutting head

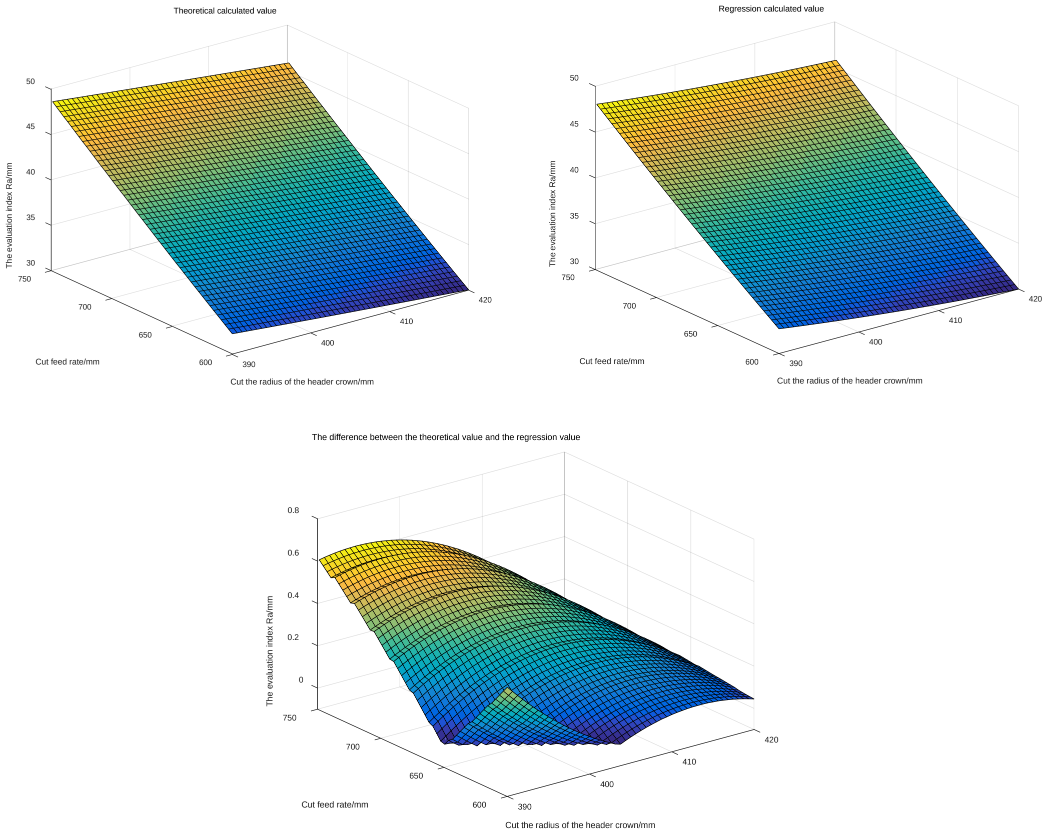

(b) "spherical crown + conical" type cutting head

Fig 4 Different cutting feed and cutting crown radius

5 Different cutting feed and cutting head conical Angle

Table 5 Basic parameter setting

"spherical crown + conical" type			
A/°	40	C/mm	400
B/mm	(600，750)	D/°	(70，85)

The characteristic evaluation index results obtained through theoretical calculation and regression calculation are shown in Fig.5.

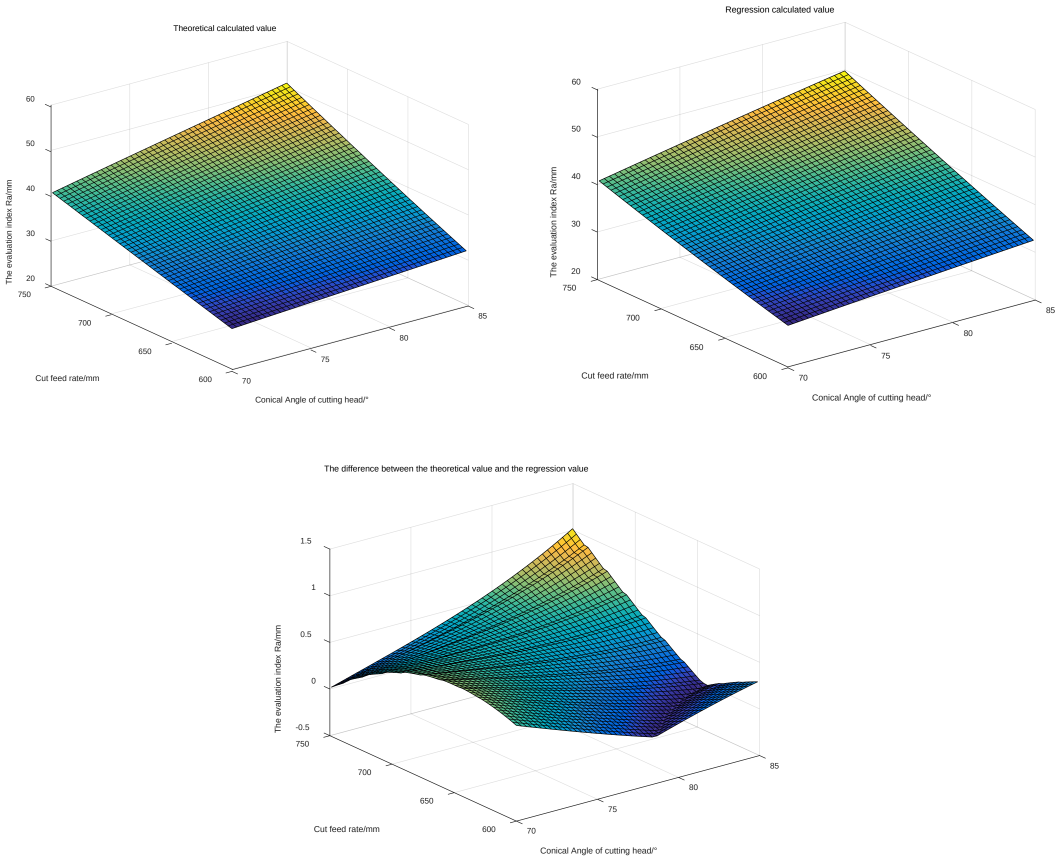

(a) "spherical crown + conical" type cutting head

Fig 5 Different cutting feed and cutting head conical Angle

6 Different cutting head crown radius and cutting head conical Angle

Table 6 Basic parameter setting

"spherical crown + conical" type			
A/°	40	C/mm	(390，420)
B/mm	700	D/°	(70，85)

The evaluation index results obtained through theoretical calculation and regression calculation are shown in Fig.6.

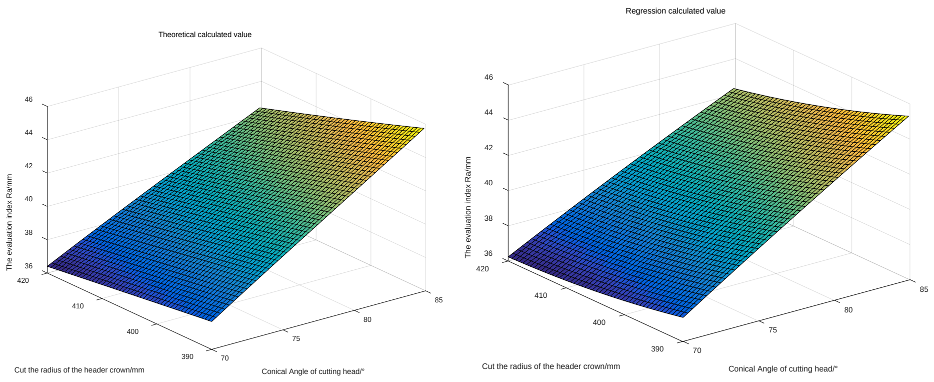

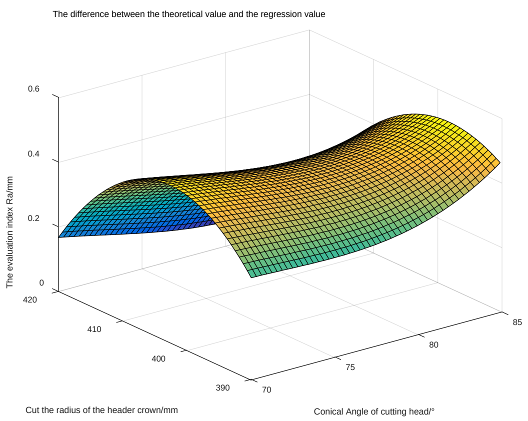

(a) "spherical crown + conical" type cutting head

Fig 6 Different cutting head crown radius and cutting head conical Angle
